# Temporal Trends in the South African Diagnostic Radiology Workforce (2002-2019)

**DOI:** 10.7759/cureus.27148

**Published:** 2022-07-22

**Authors:** Nokulunga Mkhize, Ritika Tiwari, Usuf Chikte, Richard Pitcher

**Affiliations:** 1 Division of Radiodiagnosis, Department of Medical Imaging and Clinical Oncology, Faculty of Medicine and Health Sciences, Stellenbosch University, Cape Town, ZAF; 2 Department of Radiology, Tygerberg Hospital, Cape Town, ZAF; 3 Division of Public Health and Health Systems, Department of Global Health, Faculty of Medicine and Health Sciences, Stellenbosch University, Cape Town, ZAF

**Keywords:** universal healthcare, low- to middle-income countries, imaging modalities, temporal trends, healthcare disparities, diagnostic imaging, health workforce policy, radiology

## Abstract

Background

To facilitate imaging resource planning and address key health targets of the United Nations (UN) 2030 Sustainable Development Goals, accurate data are required on imaging personnel at the country level. Such data are currently limited.

Objectives

This study aims to analyze trends in the number, geographical distribution, and demographics of South African (SA) diagnostic imaging personnel between 2002 and 2019.

Method

A retrospective analysis of the Health Professions Council of South Africa (HPCSA) database of imaging personnel from 2002 to 2019 was done. The total number of personnel and personnel per million people were calculated for the country and for each professional group (radiologist, diagnostic radiographer, and sonographer) by calendar year, province, and demographic profile. Population data were provided by Statistics SA.

Results

The total imaging personnel, number per million people, and national population increased by 283% (3,095 versus 8,753), 119% (68 versus 149/10^6^), and 29% (45.45 versus 58.77/10^6^), respectively.

Diagnostic radiographers constituted more than 80% of the workforce throughout the review period, increasing by 185% (2,540 versus 7,242). Sonographers, the smallest cohort, recorded the highest (49 versus 503; 906%) and radiologists (506 versus 1,007; 99%) the lowest proportional growth.

Although radiologists showed persistent male predominance, the male proportion decreased from 82% to 69%, while that of females increased from 18% to 31%. The average annual percentage increase in female radiologists (14%) was more than three times that of males (4%).

Diagnostic radiographers showed female predominance, but the proportion decreased from 90% to 83%, while that of males increased from 10% to 17%. Sonographers showed overwhelming female predominance (94% versus 92%). The average annual percentage increase in male diagnostic radiographers (21%) was more than double that of females (9%).

In 2002, 48% (n = 1,475) of imaging personnel identified as White, and 15% (n = 467) identified as Black African. By 2019, those identifying as White and Black African were 36% (n = 3,122) and 35% (n = 3,045), respectively.

The Western Cape Province (WCP) maintained the highest overall number of imaging personnel per million people (165 versus 233/10^6^) and Limpopo the lowest (12 versus 54/10^6^). However, Limpopo recorded the highest proportional growth in imaging personnel/10^6^ people (368%) and the WCP the lowest (41%). The differential between the best- and least-resourced provinces thus decreased from 14:1 in 2002 to 4:1 in 2019.

Conclusion

In the review period, the SA imaging workforce has shown substantial expansion and transformation and has assumed a more equitable distribution.

## Introduction

The introduction of X-rays in 1896 transformed global medical practice [1]. A range of imaging modalities has since evolved, including fluoroscopy (1899), angiography (1923), mammography (1930), ultrasound (1958), computed tomography (CT) (1970), magnetic resonance imaging (MRI) (1980), and digital X-rays (1990). Clinicians and administrators alike acknowledge that radiological services are a critical component of the healthcare system, serving as an indispensable diagnostic tool beyond the stethoscope and achieving unsurpassed anatomic resolution and specificity [2]. Notwithstanding the proven benefits of radiological services, a 2010 World Health Organization (WHO) report estimated that two-thirds of the world population, or 3.5-4.7 billion people, lack access to the most basic diagnostic imaging [3]. This may be a consequence of the many years that medical imaging was overlooked at the global health policy and planning level [4]. It is noteworthy that the first WHO publication with a specific focus on radiological services appeared as recently as 1995 [5]. In 1999, the first formal WHO policy document declared that imaging was crucial for hospitals and clinics, irrespective of location and size [6,7]. Some suggest that global health advocacy for diagnostic imaging remains muted, as evidenced by the explicit exclusion of the word “technologies” such as X-ray and ultrasound from the health targets of the United Nations (UN) 2030 Sustainable Development Goals (SDGs) adopted in 2015. Stakeholder groups, including the global Diagnostic Imaging, Healthcare IT, and Radiation Therapy Trade Association (DITTA) believe that SDG 3.8 should have read as “Achieve universal health coverage (UHC), including financial risk protection, access to essential health care services, and access to safe, effective, and affordable essential medicines, *technologies* and vaccines *and access to health* for all *people of all ages, with particular attention to the most marginalized and vulnerable populations and address antimicrobial resistance*” [8,9] and SDG 3.9b as “Support research and development of vaccines and medicines, and *technologies for health* and *functioning*, for the communicable and *non-communicable* diseases that primarily affect developing countries, provide access to affordable essential medicines, and support developing countries’ use of trade-related aspects of intellectual property (TRIPS) flexibilities” [8,9]. However, the final draft of the SDG health targets did not include the aforementioned italicized texts [10].

Additionally, global and local analyses of human resources for health typically focus on primary care needs. Particular attention is paid to nursing resources and district clinical specialist clusters (DCSCs) comprising just four specialists, namely, family physician, obstetrician, pediatrician, and anesthetist. According to the National Health Act of 2003 of South Africa (SA) and entrenched in the 2012 government publication on decentralized service delivery, DCSCs have no medical imaging personnel of any description [11]. The recognition of the pivotal role of diagnostic imaging in the healthcare system and the provision of adequate radiological equipment and personnel are important for addressing the SDG health targets and for the successful implementation of National Health Insurance (NHI).

To facilitate imaging resource planning, accurate data are required on the current quantity and distribution of equipment and personnel at the country level. There have been only limited analyses of human resources for diagnostic imaging worldwide, including in South Africa (SA) [12-14]. The rudimentary analyses to date have tended to focus on radiologist resources, with scant mention of other key imaging personnel, such as diagnostic radiographers and sonographers [13,15]. Furthermore, to the best of our knowledge, there have been just four published analyses of temporal trends in imaging personnel at the country level. Over a 16-year period (1995-2011) in the United States (US), the total radiologist cohort and the number/10^6^ people increased by 39% (27,906 versus 38,857; 2.4% per annum) and 7.5% (106.2 versus 114.2; 0.5% per annum), respectively. There was high interstate variation, with the best-resourced states exceeding the least-resourced states by a factor of more than four [16]. An Australian survey of practicing radiologists recorded an overall growth of 75% (1,150 versus 2,013; 5% per annum) between 2000 and 2016, equating to 87 radiologists per million people in 2016 [17]. Between 2014 and 2019, the total number of United Kingdom (UK) radiologists increased by 26% (3,239 versus 4,076; 5% per annum), equivalent to 56 radiologists per million people in 2019 [18]. Between 2010 and 2021, the number of diagnostic radiographers in the UK increased by 61% (23,200 versus 37,300; 5.5% per annum), reflecting 567 diagnostic radiographers at the end of the review period [14]. In Lithuania, between 2000 and 2011, the radiographer cohort decreased by 10% (964 versus 869; 1%/ per annum), although the number of radiographers per million people (27/106) did not change due to a corresponding decrease in the national population [19].

This work represents the first analysis of the temporal trends in imaging personnel at the country level in Southern Africa. Although the Health Professions Council of South Africa (HPCSA) has collected medical imaging personnel data over the decades, these have not been systematically analyzed. An accurate report of trends in the SA diagnostic imaging workforce, including radiologists, diagnostic radiographers, and sonographers, will serve as an enabling tool for future medium-term strategic planning.

The objective of this study was to analyze trends in the number, geographical distribution, and demographic characteristics of South African diagnostic imaging personnel for the period 2002-2019.

## Materials and methods

This was a retrospective, descriptive, cross-sectional study. The comprehensive national database of the HPCSA was interrogated. The database is updated annually and is the official record of all registered South African healthcare professionals. The data include the official qualifications and designated professional category of all registered healthcare workers, their addresses, and their demographic profiles. The de-identified database was procured by the Department of Global Health in the Faculty of Medicine and Health Sciences of Stellenbosch University through a formal written request to the HPCSA. All analyses were on de-identified data. Data pertaining to qualified, registered imaging professionals in the categories diagnostic radiologist, diagnostic radiographer, and sonographer for the 17-year period from January 1, 2002, to December 31, 2019, were included. Radiologists are medical doctors who have specialized in diagnostic imaging and hold a formal four-year postgraduate qualification in the interpretation of a broad range of radiological images. Radiographers are imaging technologists with a diploma or degree in the acquisition of general radiological images. Sonographers are imaging technologists with a degree or diploma in the acquisition and interpretation of ultrasound images.

Data were captured on a customized spreadsheet and stratified by a professional group, calendar year, province, and demographic profile, including gender and population group. Data were analyzed using the Statistical Package for the Social Sciences (SPSS) version 22.0 (IBM Corp., Armonk, NY, USA). Calculations were arithmetic. Data on the age profile of all categories of registered imaging personnel were collated for 2019 alone. Medical personnel in professional categories outside of diagnostic imaging and all personnel in training were excluded from the analysis.

Population data were obtained from Statistics SA. Midyear population estimates for 2002 and 2019 were imported for the whole country and the nine SA provinces. The profiling of the ethnic groups was according to the Census stratification of population groups. The four categorized groups are Black African, Colored, Asian (including Indian), and White [20].

Based on the national census figures, the number of radiologists, radiographers, and sonographers per million people was defined for each calendar year under review for the whole country and for each province. Tables expressed descriptive statistics as absolute numbers, means, and proportions.

Imaging personnel data were compared to published international data on trends in the diagnostic imaging workforce. The comparative evaluation for sonographers was limited due to the absence of compatible data.

Ethical approval and request for waiver of informed consent for the study were obtained from the Stellenbosch University Health Research Ethics Committee (reference number: S20/03/074). The project was fully endorsed by the Health Professions Council of South Africa.

## Results

National overview

During the 17-year review period (2002-2019), the number of registered SA imaging personnel almost tripled (3,095 versus 8,753; 183%) (Table 1) compared to the 29% national population growth (45.5 versus 58.8/10^6^) (Table 2) [21,22].

**Table 1:** South African diagnostic imaging personnel by calendar year, demographics, and provincial profile

	2002 (n (%))	2019 (n (%))	Total increase (n)	Average annual increase (n)	Total % increase	Average annual % increase
All imaging personnel						
Radiographers	2,540 (82)	7,242 (83)	4,702	277	185	10
Radiologists	506 (16)	1,007 (12)	501	29	99	6
Sonographers	49 (2)	504 (6)	455	27	906	50
Total	3,095	8,753	5,658	333	183	10
Overall gender profile						
Female	2,418 (78)	6,767 (77)	4,349	256	179	11
Male	677 (22)	1,986 (23)	1,309	77	193	11
Overall population group profile						
White	1,475 (48)	3,122 (36)	1,647	97	112	7
Black	467 (15)	3,045 (35)	2,578	152	552	32
Asian	268 (9)	1,004 (11)	736	43	275	16
Colored	300 (10)	946 (11)	646	38	215	13
Not specified	585 (19)	636 (7)	51	3	9	1
Overall provincial profile						
Gauteng	1,137 (37)	3,082 (35)	1,945	114.4	171	10
Western Cape	713 (23)	1,598 (18)	885	124.1	124	7
KwaZulu-Natal	511 (17)	1,556 (18)	1,045	61.5	205	12
Eastern Cape	245 (8)	684 (8)	439	25.8	179	11
Free State	169 (5)	521 (6)	352	20.7	208	12
Mpumalanga	76 (2)	398 (5)	322	18.9	423	25
Limpopo	67 (2)	322 (4)	255	15.0	381	22
North West	83 (3)	298 (3)	215	12.6	259	15
Northern Cape	34 (1)	174 (2)	140	8.2	412	24
Not specified	60 (2)	120 (1)	60	3.5	100	6
Radiologists						
Gender profile						
Male	414 (82)	693 (69)	279	16	67	4
Female	92 (18)	314 (31)	222	13	241	14
Population group profile						
White	354 (70)	593 (59)	239	14	68	4
African	14 (3)	126 (13)	112	7	800	47
Asian	45 (9)	163 (16)	118	7	262	15
Colored	2 (0)	24 (2)	22	1	1,100	65
Not specified	91 (18)	101 (10)	10	<1	11	1
Provincial profile						
Gauteng	216 (43)	439 (44)	223	13.1	103	5.7
Western Cape	125 (25)	234 (23)	109	6.4	87	4.8
KwaZulu-Natal	70 (14)	150 (15)	80	4.7	114	6.3
Eastern Cape	28 (6)	49 (5)	21	1.2	75	4.2
Free State	17 (3)	37 (4)	20	1.2	118	6.6
Mpumalanga	11 (2)	19 (2)	8	0.5	73	4.1
Limpopo	5 (1)	15 (1)	10	0.6	200	11.1
North West	3 (1)	6 (1)	3	0.2	100	5.6
Northern Cape	2 (0)	6 (1)	4	0.2	200	11.1
Not specified	29 (6)	52 (5)	23	1.4	53	2.9
Sonographers						
Gender profile						
Female	46 (94)	465 (92)	419	25	911	54
Male	3 (6)	39 (8)	36	2	1,200	71
Population group profile						
White	24 (49)	190 (38)	166	9.8	692	38.4
African	6 (12)	130 (26)	124	7.3	2067	114.8
Asian	7 (14)	97 (19)	90	5.3	1,286	71.4
Colored	4 (8)	64 (13)	60	3.5	1,500	83.3
Not specified	8 (16)	23 (5)	15	0.9	187	10.4
Provincial profile						
KwaZulu-Natal	20 (41)	149 (30)	129	7.6	645	35.8
Western Cape	14 (29)	117 (24)	103	6.1	735	40.8
Gauteng	10 (21)	162 (33)	152	8.9	1520	84.4
Eastern Cape	2 (5)	17 (4)	15	0.9	750	41.7
Limpopo	1 (3)	6 (2)	5	0.3	500	27.8
Northern Cape	1 (3)	7 (2)	6	0.4	600	33.3
Free State	0 (0)	9 (2)	9	0.5		
Mpumalanga	0 (0)	11 (3)	11	0.7		
North West	0 (0)	18 (4)	18	1.1		
Not specified	1 (3)	8 (2)	7	0.4	700	41.1
Radiographers						
Gender profile						
Female	2,280 (90)	5,988 (83)	3,708	218.1	163	10
Male	260 (10)	1,254 (17)	994	58.5	382	22
Population group profile						
White	1,097 (43)	2,339 (32)	1,242	73.1	113	7
African	447 (18)	2,789 (39)	2,342	137.7	524	31
Colored	294 (12)	858 (12)	564	33.2	192	11
Asian	216 (9)	744 (10)	528	31.1	244	14
Not specified	486 (19)	512 (7)	26	1.5	5	<1
Provincial profile						
Gauteng	911 (36)	2,481 (35)	1,570	92.4	172	9.6
Western Cape	574 (36)	1,247 (36)	673	39.6	117	6.5
KwaZulu-Natal	421 (17)	1,257 (18)	836	49.2	199	11.1
Eastern Cape	215 (9)	618 (9)	403	23.7	187	10.4
Free State	152 (6)	475 (7)	323	19	213	11.8
North West	80 (4)	274 (4)	194	11.4	243	13.5
Mpumalanga	65 (3)	368 (4)	303	17.8	466	25.9
Limpopo	61 (3)	301 (5)	240	14.1	393	21.8
Northern Cape	31 (1)	161 (3)	130	7.6	419	23.3
Not specified	30 (1)	60 (1)	30	1.8	100	5.9

**Table 2:** South African population stratified by province and calendar year * Population reduction

Province	2002 (n (%))	2019 (n (%))	Total increase (n)	Average annual increase (n)	Total % increase	Average annual % increase
Gauteng	8,170,386 (18)	15,176,116 (26)	7,005,730	412,101	85.8	5
KwaZulu-Natal	9,308,565 (20)	11,289,086 (19)	1,980,521	116,501	21.3	1.3
Western Cape	4,321,844 (10)	6,844,272 (12)	2,522,428	148,378	58.4	3.4
Eastern Cape	7,158,843 (16)	6,712,276 (11)	-446,567	-26,268	-6	-0.4*
Limpopo	5,857,622 (13)	5,982,584 (10)	124,962	7,350	2.1	0.1
Mpumalanga	3,181,041 (7)	4,592,187 (8)	1,411,146	100,796	44.4	2.6
North West	3,686,053 (8)	4,027,160 (7)	341,107	20,065	9.3	0.5
Free State	2,878,993 (6)	2,887,465 (5)	8,472	498	0.3	<0.1
Northern Cape	890,864 (2)	1,263,875 (2)	373,011	21,941	41.9	2.5
Total	45,454,211	58,775,021	13,320,810	783,577	29.3	1.7

Furthermore, the proportion of imaging personnel in the formal labor sector doubled from 0.04% (3 095/7.034 x 10^6^ people) to 0.8% (8 753/11.331 x 10^6^ people) [23,24].

Diagnostic radiographers constituted more than 80% (82% versus 83%) of the national imaging workforce throughout the review period, increasing by a factor of 2.85 (2,540 versus 7,242; 185%). Although sonographers were the smallest cohort, they recorded the highest proportional growth, with numbers increasing more than 10-fold, (49 versus 503; 906%) and advancing from 2% to 6% of all imaging personnel. Despite radiologist numbers almost doubling (506 versus 1,007; 99%), this group recorded the smallest proportional growth and decreased from 16% to 12% of the national imaging labor force.

The number of imaging personnel per million people more than doubled (68 versus 149/10^6^; 119%) (Table 3), with diagnostic radiographers showing the greatest numerical increase (56 versus 123/10^6^; 120%) and sonographers the largest proportional increase (1 versus 9/10^6^; 682%). In 2002, there were one sonographer/10^6^ people for every 10 radiologists and 55 diagnostic radiographers. By 2019, this ratio had changed to one sonographer for every two radiologists and 14 radiographers/10^6^ people.

**Table 3:** South African diagnostic imaging personnel per million population (units/10^6)

	2002 (n/10^6^ people)	2019 (n/10^6^ people)	Total increase (n/10^6^ people)	Average annual increase/10^6^ people	Total % increase/10^6^ people	Average annual % increase/10^6^ people
All imaging personnel						
Radiographers	55.9	123.2	67.3	4	120.4	7.1
Radiologists	11.1	17.1	6	0.3	54.1	3.2
Sonographers	1.1	8.6	7.5	0.4	681.8	40.1
Total	68.1	148.9	80.8	4.8	118.6	14.5
All imaging personnel by province						
Western Cape	165	233.3	68.4	4	41.5	2.4
Gauteng	139.2	202.9	63.8	3.8	45.8	2.7
KwaZulu-Natal	54.9	137.7	82.8	4.9	150.8	8.9
Free State	58.7	180.4	121.7	7.2	207.3	12.2
Eastern Cape	34.2	101.8	71.5	4.2	235.9	13.9
Mpumalanga	23.9	86.6	62.7	3.7	262.3	15.4
Northern Cape	38.2	137.5	99.4	5.8	260.8	15.3
North West	22.5	74	51.5	3	228.9	13.5
Limpopo	11.4	53.8	42.3	2.5	367.8	21.6
Most:least	14 to 1	4 to 1				
Radiologists by province						
Western Cape	28.9	34.1	5.2	0.3	18	1.1
Gauteng	26.4	28.9	2.5	0.1	9.5	0.6
KwaZulu-Natal	7.5	13.2	5.7	0.3	76	4.5
Free State	5.9	12.8	6.9	0.4	117	6.9
Eastern Cape	3.9	7.3	3.4	0.2	87.2	5.1
Mpumalanga	3.5	4.1	0.6	0	17.1	1
Northern Cape	2.2	4.7	2.5	0.1	113.6	6.7
North West	0.8	3.4	2.6	0.2	325	19.1
Limpopo	0.9	2.5	1.6	0.1	177.8	10.5
Most:least	36 to 1	14 to 1				
Radiographers by province						
Western Cape	132.8	182.2	49.4	2.9	37.2	2.2
Gauteng	111.5	163.4	51.9	3.1	712.9	41.9
KwaZulu-Natal	45.2	111.3	66.1	3.9	146.2	8.6
Free State	52.8	164.5	111.7	6.6	211.6	12.4
Eastern Cape	30	92.1	62	3.6	206.7	12.2
Mpumalanga	20.4	80.1	59.7	3.5	292.7	17.2
Northern Cape	34.8	127.3	92.5	5.4	265.8	15.6
North West	21.7	68	46.3	2.7	213.4	12.6
Limpopo	10.4	50.3	39.9	2.3	383.7	22.6
Most:least	13 to 1	4 to 1				
Sonographers by province						
Western Cape	3.2	17.1	13.8	0.8	424.7	25
Gauteng	1.2	10.6	9.4	0.6	768.9	45.2
KwaZulu-Natal	2.2	13.2	11.1	0.7	514	30.2
Free State	0	3.12	-	-	-	-
Eastern Cape	0.3	2.5	2.3	0.1	803.6	47.3
Mpumalanga	0	2.4	-	-	-	-
Northern Cape	1.1	5.5	4.4	0.3	394.6	23.2
North West	0	4.5	-	-	-	-
Limpopo	0.2	1	0.8	0	488.2	28.7
Most:least	16 to 1	17 to 1				

Gender profile

Females represented just over three-quarters (78% versus 77%) of all imaging personnel throughout the review. However, the various personnel categories displayed distinct trends. Although there was male predominance among radiologists from beginning to end (2002-2019), the male proportion decreased from 82% to 69%, while that of females almost doubled (18% versus 31%). The average annual percentage increase in female radiologists (14%) was more than three times that of males (4%).

Conversely, diagnostic radiographers demonstrated consistent female predominance, but the proportion of females decreased from 90% to 83%, while that of males almost doubled (10% versus 17%). The average annual percentage increase in male diagnostic radiographers (21%) was more than double that of females (9%).

Among sonographers, there was overwhelming female predominance (94% versus 92%). The average annual percentage of male increase (71%) was only marginally higher than that of females (54%).

Population group profile

In 2002, almost half of all registered imaging personnel (n = 1,475; 48%) identified as White, while approximately one-fifth (n = 585; 19%) were not stratified and just over one-in-seven (n = 467; 15%) identified as Black African. By 2019, the proportion identifying as White had decreased to just over one-third (n = 3,122; 36%) and was equivalent to the proportion identifying as Black African (n = 3,045; 35%), while less than one-in-10 were not stratified (n = 636; 7%).

Personnel identifying as Black African recorded the highest proportional increase (467 versus 3,045; 552%), exceeding those identifying as White (1,475 versus 3,122; 112%) by a factor of almost five (4.9) and those identifying as Asian (268 versus 1,004; 275%) and Colored (300 versus 946; 211%) by a factor of more than two. This trend was replicated among diagnostic radiographers and sonographers. However, among radiologists, those identifying as Colored showed the greatest proportional increase (2 versus 24; 1,100%), albeit from a small numerical base, while the proportional increase among those identifying as Black African (14 versus 126; 800%) exceeded those identifying as White (354 versus 593; 68%) by a factor of more than 10 (11.8%).

Provincial profile

During the review period, there were marked variations in provincial population growth. In 2002, KwaZulu-Natal had the highest population (9.30 x 10^6^ people), followed by Gauteng (8.17 x 10^6^), the Eastern Cape (7.16 x 10^6^), Limpopo (5.86 x 10^6^), and the Western Cape (4.32 x 10^6^) [21]. By 2019, Gauteng had almost doubled its population and was the most populous province (8.17 versus 15.2 x 10^6^; 85%) [22]. Additionally, by 2019, the Western Cape was placed third, having increased its population by more than half (4.32 versus 6.84 x 10^6^; 56%), surpassing both the Eastern Cape, where the population had declined (7.16 versus 6.71 x 10^6^; 6%) and Limpopo (5.86 versus 5.98 x 10^6^; 2%) where growth was negligible. The Northern Cape (0.89 versus 1.26 x 10^6^) had the lowest population throughout but nonetheless recorded moderate growth (42%).

The Western Cape maintained the highest overall number of imaging personnel per million people (165 versus 233/10^6^) and Limpopo the lowest (12 versus 54/10^6^). However, Limpopo recorded the highest proportional growth in imaging personnel/10^6^ people (12 versus 54; 368%) and the Western Cape the lowest. As a result, the differential between the best-resourced and least-resourced provinces decreased from 14:1 in 2002 to 4:1 in 2019. In 2002, 37% of all imaging personnel were in Gauteng, serving 18% of the population. By 2019, 35% of imaging personnel were in Gauteng, serving 26% of the population. Similarly, in 2002, 2% of all imaging personnel were in Limpopo, serving 13% of the national population, while by 2019, 4% of imaging personnel were in Limpopo, serving 10% of the national population.

The Western Cape also maintained the highest number of personnel per million people in the respective professional categories. However, there was a trend toward a more equitable distribution of all categories. Between 2002 and 2019, the differential between the best-resourced and least-resourced provinces decreased from 36:1 to 14:1 for radiologists and from 13:1 to 4:1 for diagnostic radiographers. Of note, in 2002, one-third of SA provinces had no registered sonographer. By 2019, all provinces had registered sonographers, but the differential between the best and least resourced provinces remained relatively high (17:1).

Age profile

Age data were not available in 2002. In 2019, the respective professional groups showed marked differences in age profile. The largest cohort of radiologists (n = 235; 23%) was in the >65-year category, while the largest diagnostic radiographer (n = 1,235; 17%) and sonographer (n = 128; 25%) grouping was in the 30- to 34-year category (Table 4).

**Table 4:** Diagnostic imaging personnel age groups in 2019

Age	2019 (n (%))
Radiologists	
20-24	-
25-29	-
30-34	20 (2)
35-39	119 (12)
40-44	160 (16)
45-49	147 (15)
50-54	94 (9)
55-59	113 (11)
60-65	96 (10)
Over 65	236 (23)
Unknown	22 (2)
Radiographers	
20-24	500 (7)
25-29	1,495 (21)
30-34	1,235 (17)
35-39	940 (13)
40-44	681 (9)
45-49	675 (9)
50-54	483 (7)
55-59	414 (6)
60-65	372 (5)
Over 65	397 (5)
Unknown	50 (1)
Sonographers	
20-24	24 (5)
25-29	80 (16)
30-34	128 (25)
35-39	93 (18)
40-44	60 (12)
45-49	39 (8)
50-54	32 (6)
55-59	25 (5)
60-65	14 (3)
Over 65	7 (1)
Unknown	2 (0.3)
Total	504

## Discussion

This project marks the first detailed analysis of the South African medical imaging workforce and provides novel insights into the evolution of the workforce in post-apartheid, democratic SA. The findings are important for medium-term strategic planning for human resources for health (HRH) and can be seen as the first step in realizing one of the key health targets of the UN 2030 Sustainable Development Goals, particularly target 3.b, namely, to substantially increase health financing, recruitment, development, training, and retention of health workers [10]. Key findings are the expansion and transformation of the SA imaging workforce, which demonstrated proportional growth almost 10-fold greater than that of the national population (283% versus 29%).

The exponential growth in sonographers documents the emergence of an entirely new professional category of imaging personnel, with proportional growth (906%) nine times that of radiologists (99%) and five times that of diagnostic radiographers (185%). These figures reflect substantial investment in sonographer training over the past two decades. Of note, diagnostic ultrasound only entered the clinical domain 64 years ago (1958), some 63 years after the discovery of X-rays [25]. The expansion in the sonographer cohort is testimony to the key role of ultrasound in the provision of imaging services in a resource-constrained environment. Ultrasound is uniquely suited to such settings since it requires no fixed installation or radiation protection. It is affordable, safe, mobile, battery-operated, and used repeatedly on the same patient and may be portable [26]. Since 2015, a four-year Bachelor of Technology (B. Tech) Degree in Sonography has been introduced in most SA institutions offering radiography training, adopted by at least three tertiary institutions to date, namely, the Durban University of Technology, Cape Peninsula University of Technology, and University of Johannesburg. In 2018, the first cohort of diagnostic sonographers graduated from the pioneering universities [27].

Provincial data provide empirical evidence of workforce redistribution, thereby enhancing equity in the provision of national imaging services. By way of example, those provinces with the highest population growth, namely, Gauteng (85%) and Western Cape (58%), documented the lowest proportional increases in personnel per million people (45% and 41%, respectively). Conversely, provinces with the lowest population growth, namely, Eastern Cape (-6%), Free State (0.3%), and Limpopo (2%), tended to record among the highest proportional increases in personnel per million people (235%, 207%, and 368%, respectively). Additionally, provinces with the lowest overall populations (Mpumalanga, North West, Free State, and Northern Cape) recorded the highest proportional growth in imaging personnel per million people, indicative of the migration of personnel to more sparsely populated areas.

Traditional social constructs are reflected in the various categories of imaging personnel, with male predominance among radiologists and female predominance among diagnostic radiographers and sonographers [28]. However, this work provides evidence of a tendency to reverse gender stereotyping among radiologists and diagnostic radiographers. In the radiologist group, the proportional increase in females exceeded that of males almost fourfold, and in the diagnostic radiographer group, the proportional increase in males was more than twofold greater than that of females.

In 2019, national population estimates by demographic profile reflected a proportional breakdown of Black African (80.7%), Colored (8.8%), Asian (2.6%), and White (7.9%) [22]. Our findings show that diagnostic radiographers are most closely aligned with the national demographic profile, with those identifying as Black African having the largest representation (36%) and historically disadvantaged individuals representing 61% of the group. Conversely, the radiologist group is least aligned with the national demographic profile, with those identifying as White comprising well over half (59%).

To the best of our knowledge, this study provides the most comprehensive analysis to date of trends in the diagnostic imaging workforce in any setting. Of note, it is the first study to include all personnel categories in the diagnostic imaging enterprise. As such, it makes an important contribution to the discourse on human resources for health and provides key, unified, baseline data for similar studies in other environments. We have shown that the average annual percentage increase in the number of SA radiologists (6%) is more than double that of the United States (US) (2.4%) and closely aligned with that of the United Kingdom (UK) (5%) and Australia (5%). Furthermore, our data show that the average annual percentage increase in the number of diagnostic radiographers (10%) is substantially higher than that of the UK (5.5%). There are no published, comparative sonographer data.

Our data show that national imaging personnel resources are broadly aligned with World Bank country classifications by income. South Africa is a high middle-income country (by World Bank stratification) but has lower radiologist resources (17/10^6^ people) than high-income counterparts such as the US (100/10^6^), Australia (87/10^6^), the UK (56/10^6^), and the average European Union country (120/10^6^) [17,18,29]. However, SA has substantially higher resources than low middle-income countries such as Kenya (4.5/10^6^), Tanzania (1.8/10^6^), and Zimbabwe (1.7/10^6^) and low-income countries such as Uganda (1.3/10^6^) and Malawi (0.2/10^6^) [30]. Similarly, SA’s 123 diagnostic radiographers/10^6^ people is lower than that of the UK (567/10^6^) but substantially higher than that of Zimbabwe (26.3/10^6^) and Malawi (10/10^6^) [14,30].

The strengths of this study are its uniqueness, its use of official HPCSA and national population data, and its 17-year duration. It was limited by the inclusion of all imaging personnel, namely, practicing, nonpracticing, and expatriates. As such, calculations of personnel per million people may be inflated. Furthermore, since HPCSA data do not record current practice location, there may be inaccuracies relating to the distribution of personnel. Additionally, there was no stratification by healthcare sector (public versus private) and thus no analysis of inter-sectoral inequity. Future studies would be enhanced if HPCSA annual registration data included current practice location and the healthcare sector of all personnel.

## Conclusions

Historical trends yield a more comprehensive view of the changes within human resources than singular data sampling. Exhaustive data on radiographers and sonographers are required.

Patterns of growth among imaging personnel are aimed at archiving equity in gender, ethnic profile, and provincial zone; plausible advances have been made albeit at different rates. Deliberate efforts in studying the mitigating factors and shaping the constituency of the workforce are essential henceforth, considering the increasing demand and widening spectrum of imaging services. The diagnostic imaging profession may face significant challenges in the near future if these pertinent issues are not addressed at the policy, planning, and training levels. This study is therefore the benchmark for future work.

## References

[1] Scatliff JH, Morris PJ (2014). From Roentgen to magnetic resonance imaging: the history of medical imaging. N C Med J.

[2] European Society of Radiology 2009 (2010). The future role of radiology in healthcare. Insights Imaging.

[3] Mollura DJ, Mazal J, Everton KL (2013). White paper report of the 2012 RAD-AID Conference on International Radiology for Developing Countries: planning the implementation of global radiology. J Am Coll Radiol.

[4] Brederhoff J, Racoveanu NT (1982). Radiological services throughout the world. Diagn Imaging.

[5] Palmer P, Breyer B, Bruguera A (2003). Manual of diagnostic ultrasound. https://apps.who.int/iris/handle/10665/38652.

[6] Ostensen H, World Health Organization, Diagnostic Imaging and Laboratory Technology Team (2001). Diagnostic imaging: what is it? when and how to use it where resources are limited?. https://apps.who.int/iris/handle/10665/66703.

[7] World Health Organisation (2017). Global atlas of medical devices. https://apps.who.int/iris/handle/10665/255181.

[8] DITTA DITTA (2020). Sustainable Development Goals (SDGS): Request for re-inclusion of technologies under health goal 3: Target 3b. https://sustainabledevelopment.un.org/content/documents/15715ditta.pdf.

[9] (2020). United Nations: Major groups and other stakeholder’s final compilation document on SD Goals and Targets (OWG13). https://sustainabledevelopment.un.org/content/documents/4438mgscompilationowg13.pdf.

[10] (2022). United Nations: Transforming our world: The 2030 agenda for Sustainable Development. https://sustainabledevelopment.un.org/post2015/transformingourworld/publication.

[11] Voce A, Bhana R, Monticelli F, Makua M, Pillay Y, Ngubane G, Kauchali S (2013). District clinical specialist teams. S Afr Health Rev.

[12] Day C, Gray A (2007). Health and related indicators. S Afr Health Rev.

[13] (2021). Human resources for health South Africa 2030: HRH strategy for the health sector 2012/13 - 2016/17. https://www.gov.za/sites/default/files/gcis_document/201409/hrhstrategy0.pdf.

[14] Michas F (2022). Statista: Annual number of medical radiographers in the United Kingdom (UK) from 2010 to 2021. https://www.statista.com/statistics/318891/numbers-of-medical-radiographers-in-the-uk/.

[15] Wishnia J, Strugnell D, Smith A (2021). Percept: The supply of and need for medical specialists in South Africa. https://percept.co.za/wp-content/uploads/2019/10/The-supply-of-and-need-for-medical-specialists-in-SA-PERCEPT.pdf.

[16] Rosenkrantz AB, Hughes DR, Duszak R Jr (2016). The U.S. radiologist workforce: an analysis of temporal and geographic variation by using large national datasets. Radiology.

[17] (2021). The Royal Australian and New Zealand College of Radiologists: 2016 Workforce Survey Report Australia. https://www.ranzcr.com/documents/4624-2016-clinical-radiology-workforce-census-report-australia/file.

[18] Callaway M (2021). The Royal College of Radiologists: Clinical radiology UK workforce census 2018 report. https://www.rcr.ac.uk/publication/clinical-radiology-uk-workforce-census-report-2018.

[19] Vanckaviciene A, Starkiene L, Macijauskiene J (2014). Supply and demand for radiographers in Lithuania: a prognosis for 2012-2030. Eur J Radiol.

[20] (2022). Statistics South Africa: Community survey 2016. Community survey.

[21] (2022). Statistics South Africa: Mid-year estimates 2002. http://www.statssa.gov.za/publications/P0302/P03022002.pdf.

[22] (2022). Statistics South Africa: Midyear population estimate 2019. Midyear Population Estimate.

[23] (2022). Statistics South Africa: Quarterly labour force survey, quarter 4 (2019). https://www.statssa.gov.za/publications/P0211/P02114thQuarter2019.pdf.

[24] (2022). National Department of Health (NDoH): 2030 human resource for health strategy: Investing in the health workforce for universal health coverage. NDoH.

[25] WI JJ, RE JM (1956). Diagnostic use of ultrasound. Br J Phys Med.

[26] Pitcher RD (2019). The role of radiology in global health. Radiology in global health.

[27] Friedrich-Nel H, Isaacs F ( 2018). The new radiography degree programmes : four years later. S Afr Radiogr.

[28] Klaveren van M, Tijdens K, Hughie-Williams M, Martín NR (2022). An overview of women’s work and employment in South Africa, revised edition. AIAS.

[29] Delbeke D, Segall GM (2011). Status of and trends in nuclear medicine in the United States. J Nucl Med.

[30] Kawooya MG, Kisembo HN, Remedios D (2022). An Africa point of view on quality and safety in imaging. Insights Imaging.

